# 3D printed models in mandibular reconstruction with bony free flaps

**DOI:** 10.1007/s10856-018-6029-5

**Published:** 2018-02-02

**Authors:** Banaszewski Jacek, Pabiszczak Maciej, Pastusiak Tomasz, Buczkowska Agata, Kuczko Wiesław, Wichniarek Radosław, Górski Filip

**Affiliations:** 1 Department of Otolaryngology Head and Neck Surgery Poznan, University of Medical Sciences, Przybyszewskiego Street 49, 60-355 Poznań, Poland; 2 Poznan University of Technology Chair of Management and Production Engineering, Poznań, Poland

## Abstract

The aim of the study was to compare two types of mandible reconstructive operations with scapula and fibula free flaps: procedures with 3-D models from thermoplastic materials and conventional planning surgeries. 8 patients were treated due to an advanced oral cavity squamous cell carcinoma. In four patients with a mandibular defect, a physical 3-D model consisting of the reconstructed and unaffected sites was prepared for a reconstruction protocol. The 3-D models were designed based to high resolution CT scans. Assessment of comparative functionality (stability of junction, mobility, mastication ability) and cosmetics was examined in both groups, following a 8 weeks healing period with better results in group with 3D model. 3-D models for mandible and donor bones allow to obtain better functionality of restored mandible in comparison to the traditional method also significantly decreases time of the operation and allows to achieve the desired shape and esthetic effect within the 1/3 of the lower face.

## Introduction

Indications for partial and total mandibulectomy include malignancies such as squamous cell carcinomas (SCC) of mandible as well as tumors extending from other parts of the oral cavity. Benign tumors such as ameloblastoma and osteoradionecrosis, rarely lead to mandibulotomy [1]. Defect size in ablative surgery depends on extension and dimensions of the pathological defect. In effect, extensive mandibular defects cause abnormal contours of the lower one third of the face, deformation and functional disorders [2]. Three dimensional shape of the mandible, presents a serious restorative challenge [3]. CT scan based computer simulation allows for attainment of physical, three-dimensional mandibular models or fabricated models of donor site [4]. Obtained models allow for a significant decrease in surgery duration as well as improvement of aesthetics and functionality of a reconstructed organ [5].

The aim of the study was to compare and two types of mandible reconstructive operations with scapula and fibula free flaps: procedures with 3-D models from thermoplastic materials and conventional planning surgeries.

## Methods

**Fig. 1:**
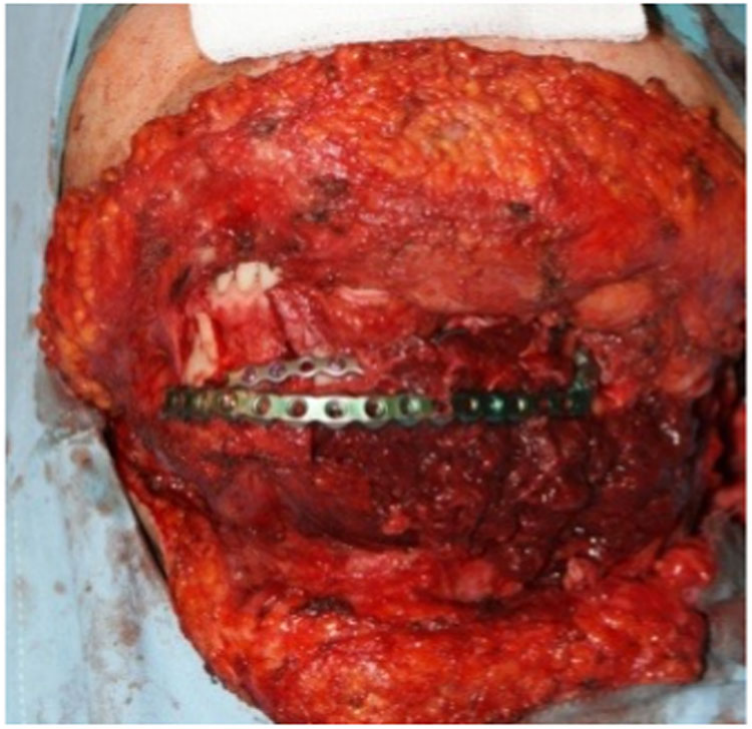
The reconstructive surgical procedure. The free fibular bone and flap is attached to the inner side of the reconstruction plate

**Fig. 2:**
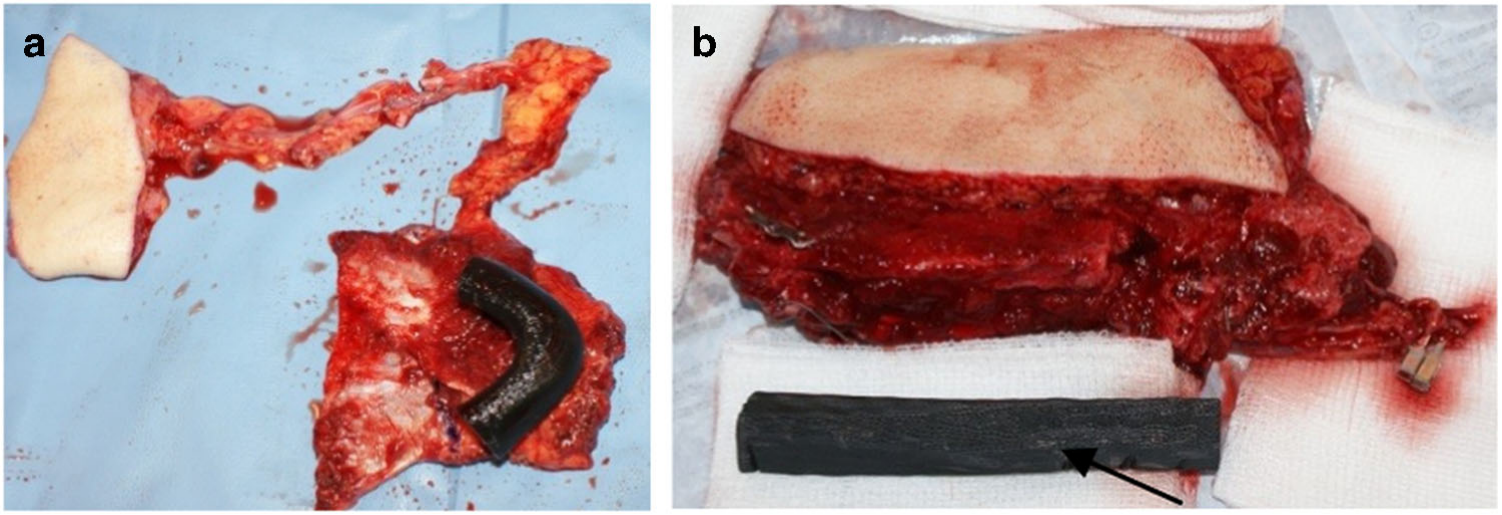
Harvesting of free osteo-cutaneous flaps: scapular with the skin island as well as 3-D model printing of the missing mandibular defect (**a**). Fibular free flap with 3D model printing. On the edge of fibula the visible directioned incisions (**b**)

8 patients were treated due to an advanced stage of oral cavity squamous cell carcinoma between 2013–2015 in the Department of Otolaryngology and Oncology, ENT Poznan University The Primary site of origin involved floor of the mouth with an extension to the alveolar ridge, body and angle of mandible. Removal of all 8 tumors, was followed by a simultaneous mandibular reconstruction (Fig. 1). Our study involved a group of 7 men and 1 women, between 49–76 years (mean age: 63.5 years median: 65 years). 6 patients were initially diagnosed with pathological changes of floor of the mouth, 2 patients with recurrence, were initially surgically treated and underwent RT/RCT. Due to an extensive spread of the lesion to the surrounding soft tissues, partial resection of the mandible in en block and dorsal part of tongue was required. Hard and soft tissue defects were reconstructed with bony free flaps, obtained from fibula (4 patients) and scapula (4 patients) with skin islands (Fig. 2). In 4 patients requiring mandibular reconstruction, a physical 3-D model was designed, which consisted of structurally unchanged elements and parts which needed reconstruction. The 3-D models were designed based to high resolution CT scans. The clinical data of the patients and a method of treatment is shown in Table 1.

**Table 1:** Clinical characterstics of the patiens treated with free osteo-cutaneous flaps

Patient No.	Pathology	Age	Gender	Lesion	TNM classification	Flap	Surgery	Method
1	SCC	61	M	Floor of the mounth Mandible (body, angle)	T4aN1M0	Scapular free flap	Segmental mandibulectomy, soft tissues	conventional
2	SCC	76	M	Floor of the mounth Mandible (body, angle)	rT4aN0M0	Scapular free flap	Segmental mandibulectomy, soft tissues	conventional
3	SCC	65	M	Floor of the mounth Mandible (body)	T4aN0M0	Fibular free flap	Segmental mandibulectomy, soft tissues	conventional
4	SCC	49	M	Submandibular gland Mandible (angle)	T4aN1M0	Fibular free flap	Segmental mandibulectomy, soft tissues	conventional
5	SCC	61	M	Floor of the mounth Mandible (body, angle)	rT4aN2M0	Fibular free flap	Segmental mandibulectomy, soft tissues	Model 3D
6	SCC	75	M	Floor of the mounth Mandible (body, angle)	T4aN0M0	Scapular free flap	Segmental mandibulectomy, soft tissues	Model 3D
7	SCC	47	M	Floor of the mounth Mandible (angle)	T4aN1M0	Fibular free flap	Segmental mandibulectomy, soft tissues	Model 3D
8	SCC	69	F	Floor of the mounth Mandible (body)	T4aN1M0	Scapular free flap	Segmental mandibulectomy, soft tissues	Model 3D

*rTNM* recurrence case, *SCC* sqamous cell carcinoma

**Fig. 3:**
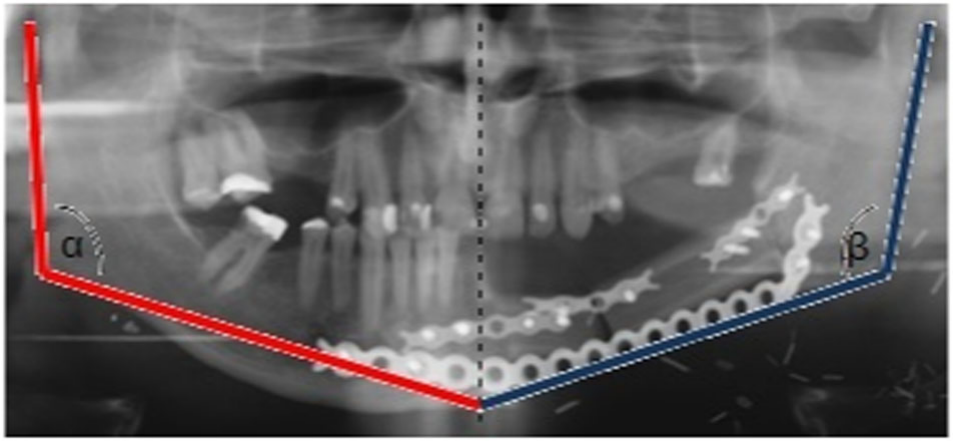
Symmetry measurement of the reconstructed mandible using image analysis. The mandibular contours from both the reconstructed (blue) and unaffected sides (red) were traced. The absolute value of the area contained between two angle α and β is defined as the differential angle (Color figure online)

**Fig. 4:**
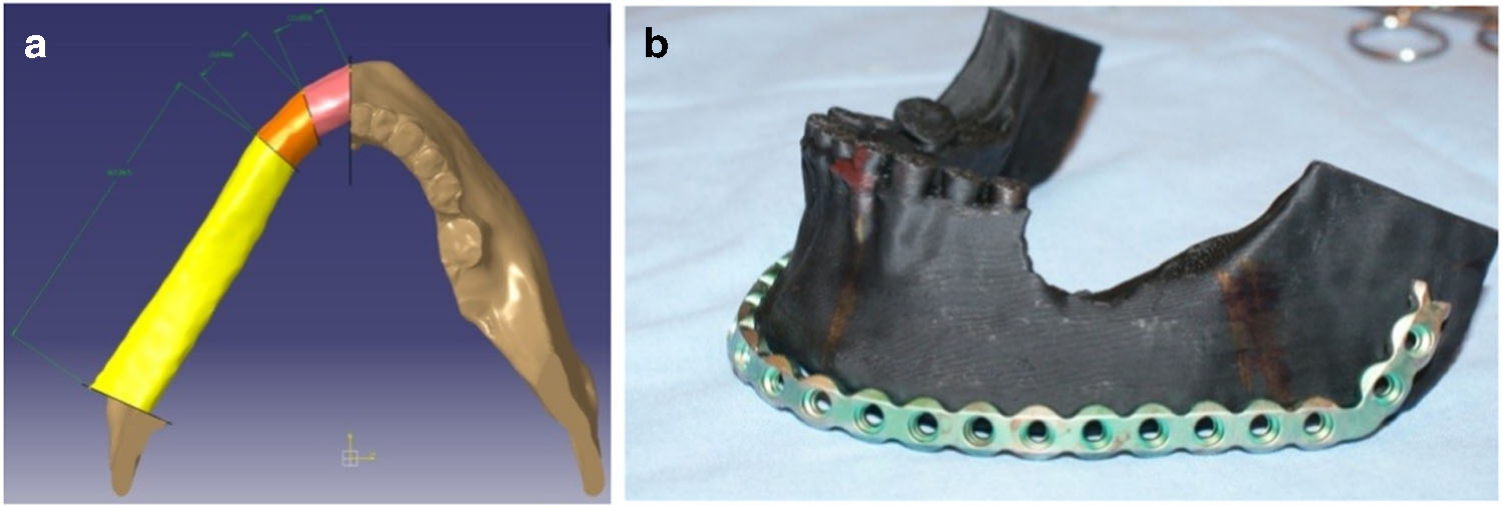
Virtual (**a**) and ready 3-D model printing of the mandible with the attached prebent reconstruction titanium plate (**b**)

Images of mandible obtained from the computer tomography were converted to digital 3-D models using the Slicer software (version 4.4.0). Created models were exported to the STL format (standard triangulation language). Raw models were then processed in the GOM Inspect v7 software, until final images of the mandible were obtained, with no deformations and redundant geometrical elements. Digital models of fibulae and scapulae were obtained and generated utilizing a similar technique. Prepared models were imported to a computer aided design system (CAD). The authors used the CATIA software. Sectioning planes were defined based upon regions of cut mandible. A piece of damaged bone was removed from the mandibular model. A missing piece was re-created through utilization of cutting planes and leading curves. The most important aspect in the process of bone filling design, was maintenance of proper shapes and determination of leading curves to obtain a desired initial shape of the mandible (Fig. 3a). Dimension BST 1200 was used to manufacture the physical models for additive manufacturing in the Fused Deposition Modelling technology. The elements of the complete lower jaw models were produced out of a thermoplastic ABS material (producer name—Dimension P400). Average manufacturing time of a lower jaw of an adult in a natural scale is approx. 5 h, with a thickness of a single layer equal to 0254 mm. Following the completion of model manufacturing, it was post-processed by removal of automatically generated supporting structures. These structures allowed for production of a model without deformation caused by gravitational deflection of plasticized material during layer deposition. According to other authors, a physical model manufactured out of ABS material in such a way has sufficient strength to use it as a functional prototype [6]. The obtained mandibular model allowed not only for preparation of an appropriate shape of titanium plates through stabilization of the bone graft with the mandible (Fig. 3b), but also provided important information for a surgeon during the operation. Prior to planned operation, Doppler of vascular pedicle of scapulae was performed and leg vessels were examined by CT angiography. Surgical outcome was determined by evaluating mandibular contour symmetry 8 weeks after the surgery. First, we traced the mandibular contours on both the reconstructed and unaffected sites. Next, we defined the absolute value of the difference between the mandibular angles as the differential angle (α and β) (Fig. 4). The values of the differential angles in the 3-D model applied during surgery and conventional groups were analyzed.

## Results

**Fig. 5:**
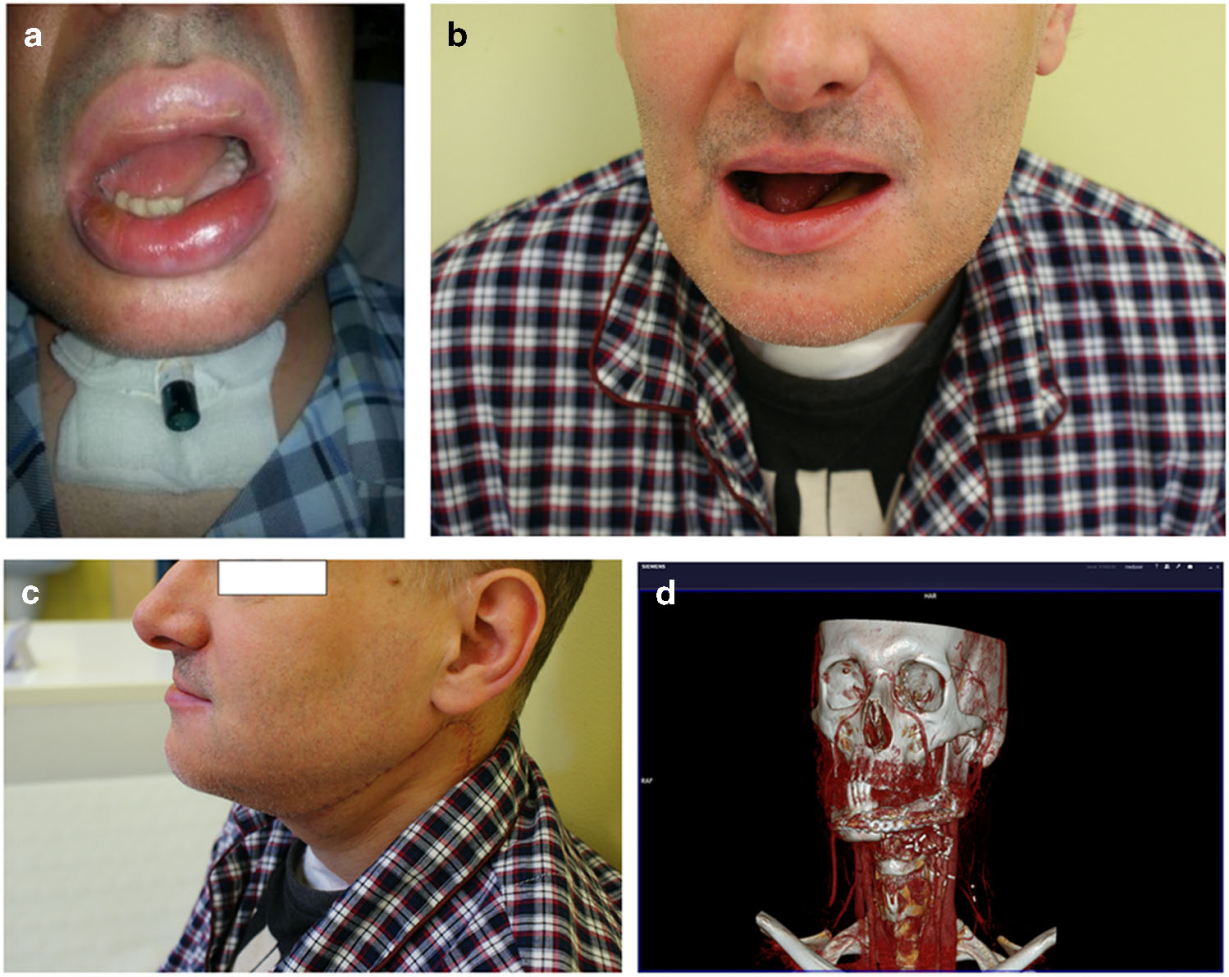
Patient following reconstructive surgery (after partial body resection of the mandible with floor of the mouth) with fibula flap using 3D model printing. **a** 1 week after surgery with fibula flap. **b** Frontal view of the patient 2 months after surgery with fibula flap. **c** Lateral view of the patient 2 months after surgery with fibula flap. **d** CT reconstruction scan

**Fig. 6:**
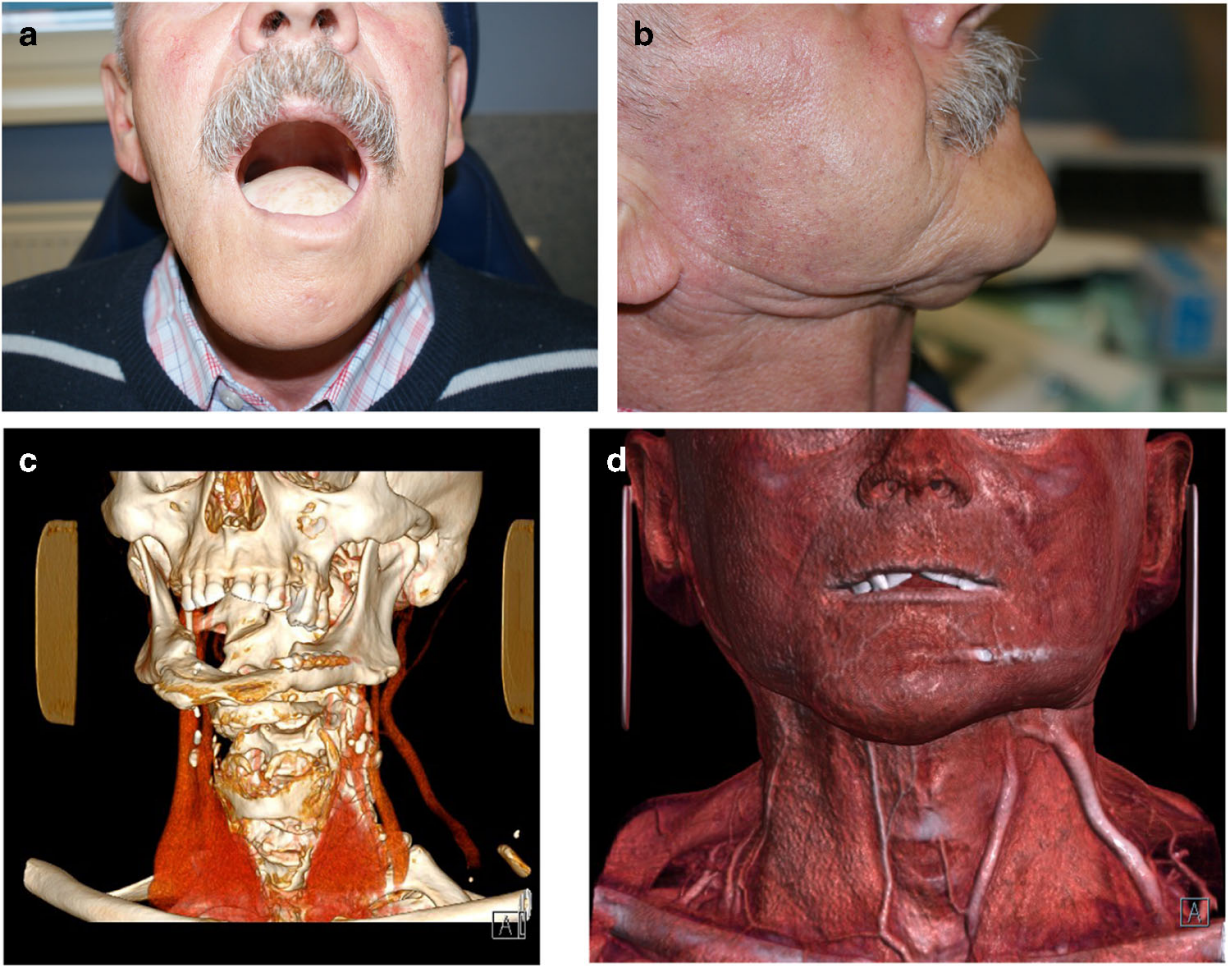
**a**–**d** Patient following reconstructive surgery with scapula chimeric flap (comlete removal of the mandibular body and tongue) using 3D model printing. **a** Frontal view 3 months after surgery with scapula chimeric flap (bone and skin island). **b** Lateral view 3 months after surgery with scapula chimeric flap (bone and skin island). **c** CT scan 3 months after surgery with scapula chimeric flap (bone and skin island). **d** CT reconstruction scan 3 months after surgery with scapula chimeric flap (bone and skin island)

All 8 procedures attained complete wound healing and were deemed as successful. Favorable healing of hard and soft tissues was attained in 4 patients in which a physical model was obtained through utilization of a three dimensional ABS technique. In 4 patients with mandibular and soft tissue defects, free fibular flap with skin island was used; a scapular flap with skin island was used for reconstruction in other four patients. The average time of surgical treatment in a group with planned 3-D model was 6.5 h vs. 8.5 h in group with the conventional plan for reconstruction. In both groups comparative evaluation functionality (junction stability, mandibular mobility, mastication ability), and cosmetics results was assessed after 8 weeks (Table 2). Differential angles were smaller in patients in which a 3D model was used (7.3 ± 9.1 degree) compared to traditional surgery (10.5 ± 12.5 degree). The mandibular contours and esthetics prior and after the surgical intervention were similar and were fully accepted by patients (Figs. 5a–d and 6a–d).

**Table 2:** Comparison of functional and aesthetic results in patients treated in conventional and 3D printing technique

Mandible funcion	Mandible reconstruction (12 chorych)	
	conventional (8 patients)	model 3D printing (4 patients)
Stability of juncion	84%	100%
Average mouth open	2.5 cm	3.0 cm
Chewing funcion	72%	90%
Acceptable cosmetics result	60%	100%
Average operation time	8.5 h	6.5 h
Mandibular contour symmetry—differental angle	10.0 ± 12.5	7.3 ± 9.1

## Discussion

The purpose of reconstructive surgery is to restore the contours of one third lower face preserve chewing function, maintain normal occlusion and provide a possibility for future dental implant placement. Proper mobility of the temporomandibular joint, allows for preservation of acceptable quality of life. Extensive defects following mandibulotomy procedure, requires reconstruction with free and pedicled vascularized bone flaps (fibula, scapula), non-vascular autologous bone graft (iliac crest, rib) or alloplastic material (titanium plate). The latter is characterized by a high rate of complications, ranging from 7–69% (plate exposure, fistulas) [7, 8]. In contrast, nonvascular bone grafts have significant limitations in reconstructive surgery; they are used in patients with a limited mandibular loss (less than 5 cm) and can lead to bone graft necrosis following radiotherapy [9].

Currently, the free vascularized flaps remain the golden standard in case of extensive mandible resection [10, 11]. It allows for reconstruction of large segmental mandibular defects. Free flaps permit a luxury of reduced time of osseointegration of the donor graft with mandible, as well as a minimal risk of bone resorption and occurrence of pathological fractures of the mandible. Additionally, flaps provide no real limitation to the patient at a later juncture in case of future radiotherapy necessitation [12]. Accurate planning of mandibular reconstructive procedure using a 3-D model printing was widely described in the literature with satisfactory esthetic and functional outcomes [13]. However, modern techniques used in reconstructive surgery require good cooperation between the radiologist, a team of engineers preparing 3-D model printing as well as the surgeons [14, 15]. The results of measured angles indicate a better mandibular symmetry in the group in which 3D model was utilized. Similar results are presented by different authors [16]. Complete stability of mandibular junction with preservation of osseointegration was confirmed during the follow-up examination, 8 weeks after the surgical intervention in all operated patients in which 3-D model printing used. It allowed for maintenance of masticatory function as well as full range of TMJ movement. 3-D model printing technique allowed for time reduction of surgical procedure by about 2.0 h, compared to typical mandibular osteotomies and reconstructions. That reduce costs of operations and reduce time of general anesthesia which have the great importance on postoperative treatment. Other authors confirmed advantages of new technique and noted the benefits to the patient due to reduced duration of anesthesia [5]. 3-D model techniques, however, require good team coordination. It can lead to a longer pre-surgical procedure compared to the traditional technique (an additional 3 days). Additionally, the use of modern technological solutions, significantly increase the costs of treatment compared to conventional reconstruction technique [5]. On the other hand rapid technological development reduces the cost of using new computer programs, 3D printing equipment and materials that make possible easer to use such techniques.

## Conclusions

1. Restoration of the mandibular continuity provides a great challenge for the reconstructing team due to its complex three-dimensional structure.
2. Applying 3-D models of the mandible manufactured using the three-dimensional printing technologies allow to obtain better functional results of a restored mandible in comparison to the traditional method.
3. The use of physical models of mandible and donor bones significantly decreases time of operation and allows for obtainment of better esthetic effect and shape of 1/3 the lower face.
